# Miltefosine treatment reduces visceral hypersensitivity in a rat model for irritable bowel syndrome via multiple mechanisms

**DOI:** 10.1038/s41598-019-49096-y

**Published:** 2019-08-29

**Authors:** Sara Botschuijver, Sophie A. van Diest, Isabelle A. M. van Thiel, Rafael S. Saia, Anne S. Strik, Zhumei Yu, Daniele Maria-Ferreira, Olaf Welting, Daniel Keszthelyi, Gary Jennings, Sigrid E. M. Heinsbroek, Ronald P. Oude Elferink, Frank H. J. Schuren, Wouter J. de Jonge, René M. van den Wijngaard

**Affiliations:** 1 Tytgat Institute for Liver and Intestinal Research, Amsterdam UMC, Location AMC, Amsterdam, The Netherlands; 2 Department of Physiology, Ribeirão Preto Medical School, University of São Paulo, São Paulo, Brazil; 3 Department of Gastroenterology and Hepatology, Amsterdam UMC, Location AMC, Amsterdam, The Netherlands; 4 Department of Neurobiology, Tongji Medical College, HUST, Wuhan, People’s Republic of China; 5 Departamento de Farmacologia, Setor de Ciências Biológicas, Universidade Federal do Paraná, Curitiba, Brazil; 6 Division of Gastroenterology-Hepatology, Department of Internal Medicine, NUTRIM School of Nutrition and Translational Research in Metabolism, Maastricht University Medical Center, Maastricht, The Netherlands; 7 Business Development, Redivia, Technische Universität, Dresden, Germany; 8 Microbiology and Systems Biology, The Netherlands Organization for Applied Scientific Research (TNO), Zeist, The Netherlands; 9 Present Address: State Key Laboratory for Infectious Disease Prevention and Control, Collaborative Innovation Center for Diagnosis and Treatment of Infectious Diseases, National Institute for Communicable Disease Control and Prevention, Chinese Center for Disease Control and Prevention, Beijing, China

**Keywords:** Antifungal agents, Fungal host response, Irritable bowel syndrome, Gastrointestinal models, Transient receptor potential channels

## Abstract

Irritable bowel syndrome (IBS) is a heterogenic, functional gastrointestinal disorder of the gut-brain axis characterized by altered bowel habit and abdominal pain. Preclinical and clinical results suggested that, in part of these patients, pain may result from fungal induced release of mast cell derived histamine, subsequent activation of sensory afferent expressed histamine-1 receptors and related sensitization of the nociceptive transient reporter potential channel V1 (TRPV1)-ion channel. TRPV1 gating properties are regulated in lipid rafts. Miltefosine, an approved drug for the treatment of visceral Leishmaniasis, has fungicidal effects and is a known lipid raft modulator. We anticipated that miltefosine may act on different mechanistic levels of fungal-induced abdominal pain and may be repurposed to IBS. In the IBS-like rat model of maternal separation we assessed the visceromotor response to colonic distension as indirect readout for abdominal pain. Miltefosine reversed post-stress hypersensitivity to distension (i.e. visceral hypersensitivity) and this was associated with differences in the fungal microbiome (i.e. mycobiome). *In vitro* investigations confirmed fungicidal effects of miltefosine. In addition, miltefosine reduced the effect of TRPV1 activation in TRPV1-transfected cells and prevented TRPV1-dependent visceral hypersensitivity induced by intracolonic-capsaicin in rat. Miltefosine may be an attractive drug to treat abdominal pain in IBS.

## Introduction

Abdominal pain is the key contributing factor to severity of IBS and, mainly due to the lack of pathophysiological insight, a major unmet clinical need^1,2^. Visceral hypersensitivity, diagnosed as a decreased threshold of discomfort to colorectal distension, is observed in ~50% of patients and thought to be an underlying mechanism for abdominal pain^3^. We recently showed intestinal mycobiome dysbiosis in hypersensitive IBS patients and addressed the possible importance of this finding in the rat maternal separation model for IBS-like visceral hypersensitivity^4^. Not only did we observe profound mycobiome dysbiosis in maternal separated rats but also provided evidence for the functional relevance of dysbiosis by conducting fecal transfer experiments in fungicide treated animals. In addition, post stress hypersensitivity to colorectal distension was reversed by blocking host recognition of particulate β-glucans. Innate immune cells recognize these fungal cell wall components via the C-type lectin receptor dectin-1 that signals via spleen tyrosine kinase (Syk). The *in vivo* use of soluble β-glucans that antagonize dectin-1 activation and a Syk inhibitor independently inhibited the pain response. Subsequent *ex vivo* experiments then indicated that particulate β-glucans trigger mast cell degranulation. Earlier studies in the maternal separation model already showed that mast cell degranulation and subsequent histamine receptor-1 activation are key events in post stress visceral hypersensitivity^5,6^. A role for mast cells in IBS was confirmed some years ago^7–9^. More recently, Wouters *et al*. used the histamine receptor-1 antagonist ebastine to reduce visceral hypersensitivity and abdominal pain in IBS patients^10^. In the same investigation, live calcium imaging experiments with patient rectal biopsies suggested that the underlying mechanism involves histamine receptor-1 mediated sensitization of TRPV1. Indeed, an important role for this nociceptive ligand-gated cation channel was already predicted in the maternal separation model, where post stress visceral hypersensitivity was reversed by a specific TRPV1 antagonist^6^.

Taken together, the above data suggest that post stress visceral hypersensitivity results from immune recognition of an aberrant mycobiome via the dectin-1/Syk pathway, leading to mast cell degranulation and subsequent activation of histamine-1 receptors on sensory neurons, which in turn leads to sensitization of TRPV1 and pain signaling. The process of drug development for newly identified targets such as the ones described here is costly and time-consuming. Repurposing of existing drugs with established side effects may partly circumvent these issues^11^. In search for candidate drugs to treat abdominal pain complaints in IBS, we evaluated the FDA approved compound miltefosine. This alkyl-phospholipid was, largely unsuccessful, developed as an antitumor drug but is now approved as an oral treatment of visceral leishmaniasis^12,13^. Miltefosine however, was also shown to have broad-spectrum *in vitro* and *in vivo* fungicidal activity by triggering metacaspase (MCA1)-dependent apoptosis in fungal target cells^14,15^. Thus, *in vivo* administration of this compound may lead to favorable mycobiome modulation in dysbiotic subjects. Miltefosine also inhibited *in vitro* mast cell activation, and oral and topical administration were successfully evaluated in mast cell driven skin conditions^16–19^. Mast cells are modulated in the cytosol by inhibition of Ca^2+^-dependent protein kinase C (cPKC)^18^ and, due to insertion of this phosphatidylcholine analogue, at the plasma membrane were it behaves as a lipid raft modulator^13,20^. Lipid rafts are specialized membrane microdomains that are formed by tightly packed aggregates of phospholipids, glycolipids and cholesterol together with protein receptors, which can be included or excluded depending on their affinity. These rafts act as signal transduction moieties^21^. In trigeminal sensory neurons and TRPV1 transfected cell lines, disruption of raft integrity affected TRPV1 receptor activation by inhibiting opening properties of the cation channel^22^. Although raft disrupting strategies other than miltefosine were used, these result suggest that miltefosine, in addition to direct targeting of the mycobiome and mast cells, may also interfere with TRPV1 receptor activation to alleviate abdominal pain in IBS.

Here, we evaluated the effect of miltefosine treatment in two different models of visceral hypersensitivity. In the rat maternal separation model, we addressed the possible correlation between miltefosine-induced reversal of post-stress visceral hypersensitivity and fecal myco- and microbiome alterations. Reported fungicidal and bactericidal effects of miltefosine^15,23^ were confirmed with *in vitro* agar disk diffusion tests. Finally, we evaluated whether miltefosine reduces the effect of TRPV1 activation in TRPV1-transfected cells, and investigated its effect on *in vivo* TRPV1-dependent visceral hypersensitivity in a rat model of intracolonic capsaicin.

## Results

### Miltefosine treatment reverses post-stress visceral hypersensitivity in maternal separated rats

**Figure 1:**
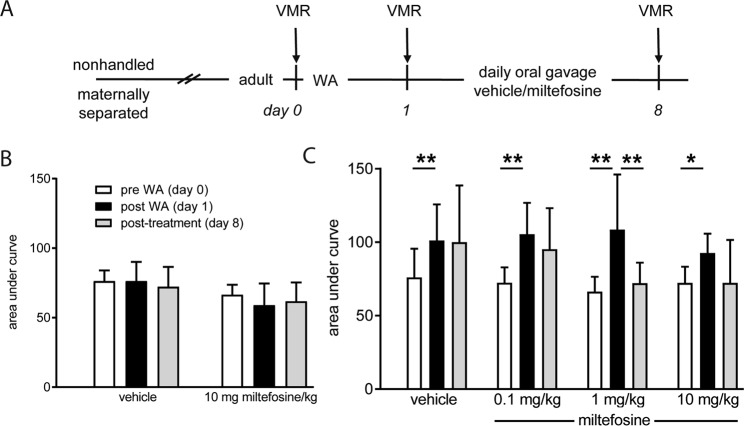
Miltefosine treatment reversed post water avoidance (WA) visceral hypersensitivity in maternally separated rats. (**A**) Schematic representation of the experimental set-up; the visceromotor response (VMR) to distension was measured before and 24 hours after WA, and after 7 day miltefosine or vehicle treatment. Data shown in histograms (**B**,**C**) reflect results of nonhandled and maternal separated rats respectively. Data are given as area under the curve of the relative response to colorectal distension. All data are mean +/− SD, n = 7–10, *P < 0.05 and **P < 0.01 (Repeated Measures one-way ANOVA, Sidak’s post hoc test).

To address the possible therapeutic potential of miltefosine, we first evaluated its effects in the rat maternal separation model (experimental setup depicted in Fig. 1A). Similar to our previous investigations^4–6,24,25^, WA-stress at adult age did not lead to enhanced visceromotor response to colorectal distension in nonhandled rats (Fig. 1B, day 0 *vs* day 1). Moreover, 7 days post-WA treatment with vehicle or miltefosine (10 mg/kg/day) did not change the visceral sensitivity status of these nonhandled animals (day 1 *vs* day 8). Compared to pre WA, all 4 maternal separation groups showed enhanced visceromotor response to distension after WA-stress (Fig. 1C, day 0 *vs* day 1). This response was reversed after 7 day treatment with 1 mg miltefosine/kg (day 1 *vs* day 8), but not by vehicle, 0.1 and 10 mg miltefosine/kg. Lack of significant reversal in the 10 mg/kg treatment group may be due to relatively low post-stress visceral sensitivity at day 1. The latter is illustrated by comparing the mean difference in area under the curve (AUC in arbitrary units) between day 0 and day 1 (*i*.*e*. pre- vs post-WA) of the 1 mg/kg and 10 mg/kg treatment groups; Δ AUC 42 and 20 respectively.

### Miltefosine inhibits *in vitro* growth of *Candida albicans* and *Bacillus subtilis*

**Figure 2:**
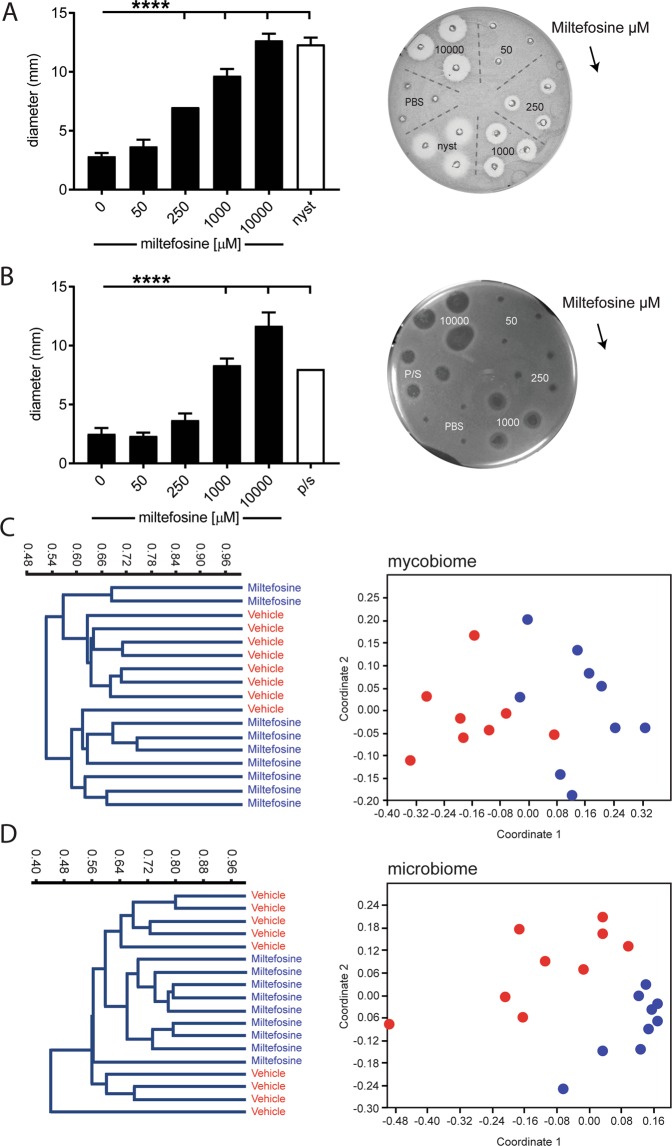
Miltefosine induced *in vitro C*. *albicans* and *B*. *subtilis* growth inhibition and *in vivo* differences in post-treatment myco- and microbiome composition. Right side photographs show agar disk diffusion assays with *C*. *albicans* (**A**) and *B*. *subtilis* (**B**). Arrows indicate direction of miltefosine concentration series (50, 250, 1000 and 10000 µM). Controls: phosphate buffered solution (PBS), nystatin (nyst) and penicillin/streptomycin (p/s). Histograms (**A**,**B**) show the average diameter of resulting halos (mean +/− SD, n = 3, ****P < 0.0001, one-way ANOVA and Dunnett’s post hoc test). Visualization of the fecal myco- and microbiome of maternally separated rats subjected to vehicle or miltefosine is shown in (**C**,**D**) respectively. The Bray-Curtis dissimilarity index was used to generate the left side dendrograms and right side non-metric multidimensional scaling plots.

Fungicidal and bactericidal activity may be relevant to the observed miltefosine-induced reversal of post-stress visceral hypersensitivity in maternal separated rats. Therefore, we carried out radial diffusion assays to confirm antibiotic activity. In *C*. *albicans* seeded agar, the inhibitory effect of nystatin justified the use of this assay as an anti-fungal readout (Fig. 2A). Compared to vehicle (i.e. phosphate-buffered saline; PBS), 250 µM, 1000 µM and 10 mM miltefosine induced a dose dependent inhibition of fungal growth. *B*. *subtilis* seeded agar was then used to evaluate possible bactericidal activity. The positive control, a penicillin/streptomycin mixture, as well as 1 mM and 10 mM miltefosine induced significant growth inhibition (Fig. 2B). Taken together, these results confirmed earlier reports on the anti-fungal and anti-bacterial activity of miltefosine^15,23^.

### Miltefosine treatment modulates the intestinal microbiota in maternal separated rats

The *in vitro* fungicidal and bactericidal properties of miltefosine prompted us to explore whether successful miltefosine treatment in the maternal separation model associated with intestinal myco- and microbiome differences. We performed high-throughput rDNA sequencing of fungal ITS-1 and bacterial ribosomal 16S genes. Amplicons were generated with DNA isolated from fecal pellets of vehicle treated and miltefosine (10 mg/kg/day) treated maternal separated rats, obtained on day 8 post treatment. One DNA sample of the vehicle group did not generate sufficient amount of sequencing reads for mycobiome analysis.

Hierarchical clustering based on the Bray-Curtis dissimilarity index and the UPGMA algorithm was performed on classified fungal species. The resulting dendrogram (left panel Fig. 2C) showed two main clusters. The upper cluster contained 7 (out of 8) vehicle treated and 2 (out of 9) miltefosine treated maternally separated rats. The lower cluster contained 1 vehicle treated and 7 miltefosine treated maternally separated rats. Similar results were obtained by non-metric multidimensional scaling (right panel Fig. 2C). Spatial patterns obtained with this ordination technique revealed two diffuse but separate clusters for vehicle and miltefosine treated rats. One way PERMANOVA multivariate statistics indicated a significant difference between groups (*p* = 0.0009, F = 6.6). To compare the bacterial microbiome of maternally separated rats treated with either miltefosine or vehicle, we again performed clustering based on the bray Curtis dissimilarity index and UPGMA algorithm. Compared to the mycobiome analysis, the resulting dendrogram (left panel Fig. 2D) showed less clear separation into treated and untreated clusters. Nevertheless, non-metric multidimensional scaling (right panel Fig. 2D) revealed differential spatial patterns for the two treatment groups. One way PERMANOVA showed significant difference between groups (*p* = 0.0001, F = 4.1). Collectively, our data suggest that miltefosine treatment modulates both the fecal myco- and microbiome.

### Miltefosine affects *in situ* mast cell staining intensity in colonic mucosa

**Figure 3:**
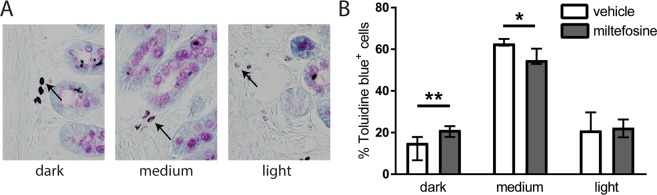
The % of intensely stained mast cells was higher in miltefosine treated tissues. Arrows in left side photographs (**A**) indicate representative examples of different mast cell staining intensities obtained with Toluidine Blue. (**B**) Shows % mucosal mast cells per staining intensity when comparing tissue sections of miltefosine and vehicle treated maternal separated rats. Data are in median & range, *P < 0.05, **P < 0.01 (Mann-Whitney U test).

Although the *in vivo* effect of miltefosine may depend on mycobiome and microbiome modulation, a direct effect on mast cells may also be relevant^16–19^. We performed Toluidine Blue stainings on colonic mucosa of vehicle and miltefosine treated maternal separated rats, and assessed differences in mast cell (granule)-staining intensity as indirect measure for *in vivo* mast cell degranulation (example stainings in Fig. 3A). Upon miltefosine treatment, we observed higher number of darkly stained mast cells and lower number of mast cells with medium staining intensity (Fig. 3B). These data suggest that post-stress mast cell degranulation was partly prevented by miltefosine treatment.

### Miltefosine affects *in vitro* TRPV1 activation by capsaicin

**Figure 4:**
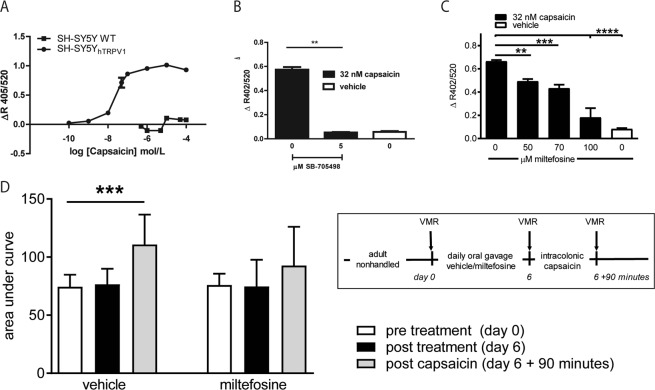
Miltefosine interfered with *in vitro* and *in vivo* capsaicin-induced TRPV1 activation. (**A**) Intracellular free calcium levels in response to different dosages of capsaicin in wildtype- and TRPV-1 transfected SH-SY5Y human neuroblastoma cells. (**B**) Capsaicin-induced activation of SH-SY5Y_hTRPV1_ cells in the presence of a specific TRPV1 antagonist (SB-705498). (**C**) Capsaicin-induced activation of SH-SY5Y_hTRPV1_ cells, pre-incubated with different dosages of miltefosine (mean +/− SD, **P < 0.01, ***P < 0.001, ****P < 0.0001, one-way ANOVA, Dunnett’s post hoc test). (**D**) Schematic representation of experiments performed in the intracolonic capsaicin model and results of colorectal distensions in this model. Results are given as area under the curve of the relative response to distension (mean +/− SD, n = 9–10, ***P < 0.001, Repeated Measures one-way ANOVA, Sidak’s post hoc test).

The TRPV-1 ion channel, sensitized via the histamine-1 receptor, is an essential nociceptor in the rat maternal separation model and IBS patients^5,6,10^. Because the opening properties of TRPV1 are lipid raft dependent, the analgesic effect of miltefosine may partly result from altered TRPV1 gating^22,26^. We first addressed this possibility in an *in vitro* model system. Comparing wildtype SH-SY5Y and TRPV1 transfected SH-SY5Y_hTRPV1_ neuroblastoma cells, we showed dose-dependent capsaicin-induced increase in intracellular Ca^2+^ levels in TRPV1 transfected but not in wildtype cells (Fig. 4A). Based on this experiment, the 32 nM capsaicin concentration was used in further investigations. To confirm strict TRPV1 dependence of the capsaicin response, SH-SY5Y_hTRPV1_ cells were then pre-incubated with SB-705498^27^. This selective TRPV1 antagonist prevented the capsaicin-induced increase of cytosolic Ca^2+^ (Fig. 4B). Next, SH-SY5Y_hTRPV1_ cells were pre-incubated with different concentrations of miltefosine, which dose-dependently decreased capsaicin-induced TRPV1 activation (Fig. 4C).

### *In vivo* TRPV1 dependent visceral hypersensitivity is prevented by miltefosine treatment

TRPV1 activation is highly relevant in post stress visceral hypersensitivity of the maternal separation model^6^. However, from the results shown in Fig. 1, it cannot be dissected whether or not TRPV1 was an *in vivo* target for miltefosine. Earlier, we administered intracolonic capsaicin to nonhandled Long Evans rats and showed that the resulting visceral hypersensitivity is strictly TRPV1 dependent^24^. Here, we first subjected normal Long Evans rats to a 1 week miltefosine treatment protocol (gavage 10 mg/kg/daily) and then applied 0.1% intracolonic capsaicin. The experimental setup of the experiment is depicted on the right side of Fig. 4D. In vehicle treated rats, capsaicin induced an enhanced response to colonic distension that was not observed when rats were pretreated with miltefosine. These findings suggest that miltefosine’s *in vivo* mode of action may involve targeting of TRPV1.

## Discussion

**Figure 5:**
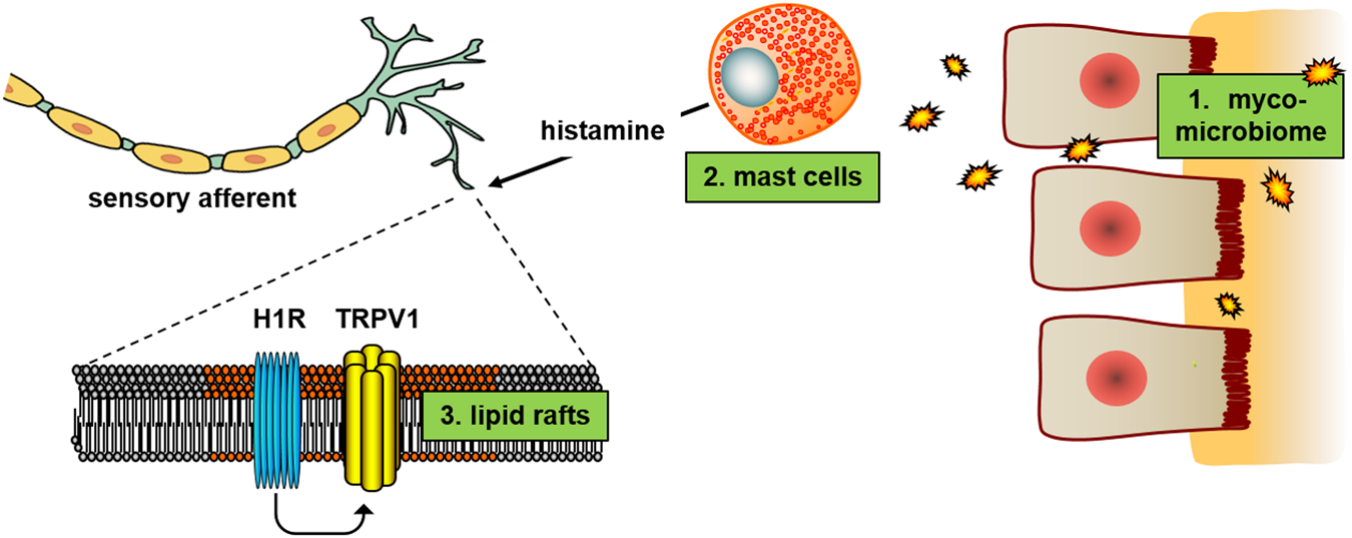
Miltefosine-targets identified in the maternal separation model. Miltefosine affected the fecal myco- and microbiome (1), mast cell degranulation (2) and the TRPV1 ion channel (3). Effects on the histamine 1 receptor (H1R) may have been relevant as well but were not addressed in the current investigations.

Treatment of IBS is challenging due to the heterogeneous nature of the disorder and, perhaps as a result thereof, lack of truly efficacious therapies. In at least part of the IBS patients, abdominal pain may arise due to immune recognition of an aberrant gut mycobiome. In response, mast cells release histamine and, via the histamine 1 receptor, sensitize TRPV1 on afferent sensory neurons leading to abdominal pain^4–6,9,10^. In search for novel treatment options, we identified miltefosine as a candidate drug because it reversed post stress visceral hypersensitivity in the IBS-like rat model of maternal separation. In follow up experiments we showed that it may have targeted several of the different mechanisms leading to fungal-induced visceral hypersensitivity (schematic overview in Fig. 5). In line with miltefosine’s fungicidal and bactericidal activity, reversal of visceral hypersensitivity associated with altered gut microbiome composition. In addition, miltefosine affected stress-induced degranulation of mucosal mast cells. Finally, this drug inhibited TPRV1 activation in TRPV1 transfected neuroblastoma cells, and prevented *in vivo* capsaicin-induced TRPV1 activation and resulting visceral hypersensitivity in normal rats. Thus, miltefosine may exert its analgesic effect by acting on 3 different levels of mycobiome-induced visceral hypersensitivity.

Similar to any other animal model, the maternal separation model in rat has its limitations when trying to mimic a complex and enigmatic disorder like IBS. Nevertheless, targets identified in earlier pre-clinical investigations, i.e. mast cells and the histamine 1 receptor, were successfully translated to human^5,6,9,10^. Therefore, we used this rat model to assess whether orally administered miltefosine, which crosses the intestinal epithelium by a non-specific passive paracellular pathway^28^, should be a drug candidate to alleviate abdominal pain in IBS. Previous investigations showed fungicide-mediated reversal of post stress visceral hypersensitivity in maternal separated rats. Maternal separated and nonhandled Long Evans rats also differed in gut mycobiome composition, and fecal transfer experiments indicated that the observed mycobiome dysbiosis was relevant for visceral hypersensitivity^4^. Thus, compounds capable of inducing mycobiome changes may also affect visceral hypersensitivity. Indeed, miltefosine treatment led to reversal of hypersensitivity while the post treatment mycobiome of miltefosine- and vehicle treated rats differed. Whether these mycobiome associations were causally relevant cannot be deduced from the current data. Yet, the fluconazole/nystatin induced reversal of hypersensitivity that we showed earlier, also associated with compositional changes of the gut mycobiome^4^. In addition, the miltefosine findings are reminiscent of results obtained with a mixture of essential oils from *Mentha* x *piperita* L. and *Carum carvi*. The main components of these oils are menthol and (+)-carvone respectively. Both components were published to have fungicidal activity which we confirmed by agar disk diffusion assays^29,30^. Indeed, when maternal separated rats were treated with the oil combination, reversal of hypersensitivity associated with a shift in mycobiome composition^31^. In parallel with these essential oil results, miltefosine treatment not only led to fungal but also bacterial microbiome changes. Because previous experiments showed an essential role for immune recognition of fungal β-glucans, we suggest that the bacterial microbiome is not the main cause for visceral hypersensitivity in these animals^4^. Alterations of the bacterial microbiome may however also affect the gut mycobiome^32^, and it cannot be excluded that initial bactericidal effects of miltefosine led to secondary but relevant mycobiome changes.

In the maternal separation model, gut mycobiome dysbiosis is essential for the activation of mast cells which eventually leads to visceral hypersensitivity^4–6^. Others have shown that miltefosine is capable of inhibiting *in vitro* mast cell activation and successfully used this compound as a therapeutic intervention strategy for mast cell mediated diseases^16–19^. In order to further expand knowledge on possible *in vivo* targets of miltefosine we mainly focused on mechanisms and cell types other than mast cells. We did perform however *in situ* Toluidine Blue stainings that suggested a lower level of degranulation in miltefosine treated rats. Whether this was due to direct targeting of mast cells, or an indirect effect via microbiome modulation cannot be concluded from this limited evaluation. One mechanism via which miltefosine may have affected mast cells directly is via insertion into lipid rafts^13,18,20^. These rafts provide the optimal microenvironment for ligand receptor interactions and subsequent recruitment of cell signaling molecules. Moreover, in case of ion channels, lipid rafts can regulate channel function^21,33^. Using a selective TRPV1 antagonist, we previously showed an important role for this afferent expressed nociceptive cation channel in post stress visceral hypersensitivity of maternal separated rats^6^. Others provided evidence that interactions between TRPV1 and lipid raft interfaces regulate its gating properties^22,26^. Indeed, our *in vitro* results showed that selective capsaicin-induced TRPV1 activation can be inhibited by miltefosine treatment. The latter suggests that miltefosine may have targeted TRPV1 in the maternal separation model as well. Unfortunately however, we are unable to assess the relative contributions, if any, of miltefosine mediated mycobiome-, mast cell- and TRPV1 modulation in the maternal separation setting. Nevertheless, results obtained with the intracolonic capsaicin model confirmed that miltefosine is capable of interfering with *in vivo* TRPV1 activation. Concerning the role of this ion channel it is important to note that histamine 1 receptor ligation leads to sensitization of TRPV1 and subsequent visceral hypersensitivity in IBS patients^10^. Because sensitization depends on intracellular signaling pathways, it can be envisaged that TRPV1 and the histamine 1 receptor translocate to the same lipid rafts for optimal interaction. Although our previous investigations showed the relevance of the histamine 1 receptor in the maternal separation model^5^, we did not address whether miltefosine also interfered with this TRPV1 sensitization mechanism.

Because miltefosine is not specifically targeting TRPV1 containing lipid rafts, signaling pathways not necessarily relevant to abdominal pain may have been affected as well. The cell membrane however, holds many different types of highly dynamic and coexisting rafts with associated proteins^34^ and miltefosine microdomain affinity may differ according to dissimilarities in composition. Although this suggests that not all raft assemblies and associated events were targeted to the same extent, unwanted side effects should be considered. During a 4 week, randomized, double-blind, placebo controlled trial, Magerl *et al*. tested the use of orally administered miltefosine in chronic spontaneous urticaria^16^. In this mast cell and histamine dependent skin condition, the urticaria-activity-score levels and number of weals were substantially more reduced in miltefosine treated patients when compared to placebo treatment. The highest daily treatment dose was 150 mg (average patient weight 84.2 kg), which resembles the 1 mg/kg dose used in the maternal separation model. Although no serious adverse events were reported, mild to moderate adverse events, including nausea and vomiting, were frequent in miltefosine and placebo treatment groups. In addition, beneficial effects of miltefosine over placebo were lost within four weeks after discontinuation of treatment. It can be envisaged that also in IBS long term miltefosine treatment may be needed for a continued effect on TRPV1 dependent pain, but this is not preferable considering the unwanted side effects. Alternatively, miltefosine may be used as a lead compound for the development of drugs effectively modifying TRPV1 responses in the absence of side effects. Yet, this contradicts our original intension of repurposing an existing drug for IBS therapy. Knowing however that miltefosine targets the gut microbiome as well, we suggest that short term treatment should be considered in order to induce a favorable reset of the mycobiome. Our previous results suggested that this can also be achieved with fungicides like fluconazole and nystatin, which are clinically used to treat fungal infections^4^. Fungal resistance against these compounds is however on the rise, and using them for a non-lethal but highly prevalent disorder like IBS may further shorten their clinical life span^35^. Since this would lead to a further increase of infection related deaths, alternative compounds like miltefosine should be considered for IBS therapy. Prior to embarking on patient studies however, the long-term persistence of miltefosine treatment-induced mycobiome changes should be monitored in a relevant preclinical setting, preferably in rats with humanized IBS gut micro/mycobiome. In case such results are sufficiently gratifying, we suggest that future clinical trials monitor post treatment symptom improvement and duration thereof, and correlate these results to persistence of mycobiome changes.

Abdominal pain in IBS is an unmet clinical need, and development of novel drugs is highly time consuming and costly. The recent identification of novel targets made it possible to evaluate an existing non-selective but FDA approved drug with known safety profile. Treatments with so-called ‘dirty drugs’ are often avoided. However, off target effects can be used in a meaningful manner, because they enable repurposing of existing therapeutic compounds to other disorders^11^. Moreover, promiscuous drugs might be more effective than single target drugs^11,36^. In an animal model with proven predictive value for IBS, we showed that miltefosine changed the gating properties of the nociceptor TRPV1 and affected mast cell activation and the gut mycobiome. Our results suggest that miltefosine should be evaluated for the treatment of abdominal pain in IBS.

## Methods

### Animals

Long-Evans rats (Harlan, Horst, The Netherlands) used for the current manuscript (n = 7–10/group), were bred at our local animal facility (Amsterdam UMC, Location AMC, Amsterdam, The Netherlands). All rats shared the same room and were kept in open cages. Research was conducted in accordance with institutional guidelines and approved by the Animal Ethical Committee of the AMC/University of Amsterdam (Reference Protocol Number 100998).

### Measurement of the visceromotor response to colonic distension and data analysis

Visceral hypersensitivity in patients is diagnosed as an increased sensitivity to rectal distension, contributing to abnormal perception of pain and discomfort^3^. During distensions, self-rating questionnaires (i.e. visual analogue score) are often used to evaluate pain scores, but these cannot be used in rat. However, due to a spinal reflex, colorectal distensions in rats will induce abdominal muscle contractions (the visceromotor response) that we quantified by electromyography (EMG) recording^37^. To quantitate the absolute response to colorectal distension, EMG data obtained during 20 second tracing periods, prior to distension and during distension, were extracted from the raw data sets, properly processed and subtracted. Next, the response to the first maximum volume distension (i.e. pre-stress or pre-capsaicin 2 mL distension) was defined as the 100% response, and all other responses of the same animal were related to this (as such, the post-stress maximum-volume response of a maternal separated rat will most likely be higher than 100%). The AUC of these relative responses was calculated for individual rats and used for statistical analyses. For extensive description and technical details of this technique and data analysis, please refer to our earlier publications^4–6,25^.

### Colonic distension protocol

Distensions were performed at adult age (≥4 months) with a latex balloon (Ultracover 8F, International Medical Products BV, Zutphen, The Netherlands) and carried out as described before^4–6,25^. In short, after insertion of the balloon-catheter under short isoflurane anesthesia, rats were allowed to recover for 20 minutes. Next, the balloon was distended with graded volumes of water (1.0, 1.5, and 2.0 mL). During maximum distension, length and diameter of the balloon were 18 and 15 mm, respectively. After each 20 sec distension period, water was quickly removed and rats were allowed to an 80 sec resting period.

### Post water avoidance (WA) miltefosine treatment in the maternal separation model

In humans, early adverse life events are associated with IBS at adult age and stress is a trigger for visceral hypersensitivity in IBS patients^38,39^. These features of IBS are mimicked in the maternal separation model where Long Evans rats are subjected to neonatal maternal separation, followed by an acute WA-stress at adult age. The combined insults were shown to result in post stress visceral hypersensitivity to colorectal distension that is not observed when nonhandled rats are subjected to WA^25^. For the current experiments, dams were separated from their pups for a period of 3 hours per day from post-natal day 2 to 14. During separation, the dam was placed in a different room while the litter remained in its own cage, placed upon a heat mat to maintain body temperature of the pups. Nonhandled control rats were nursed normally. Pups were weaned at postnatal day 22, and further experiments were carried out at a minimum age of 4 months. Distension protocols were performed pre-WA, 24 hours post-WA and post 7 days treatment. During 1 hour WA, rats were placed on a pedestal surrounded by water^4–6,25^. Miltefosine (JADO Technologies, Dresden, Germany) was dissolved in demineralized water and administered once daily by oral gavage, starting 30 minutes after the first post-WA distension protocol. Maternally separated rats received vehicle or 0.1, 1 or 10 mg miltefosine/kg daily, nonhandled rats received vehicle or 10 mg miltefosine/kg daily. A schematic representation of these experiments is given in Fig. 1A. Our dosing strategy was based on a successful clinical trial performed in patients with chronic spontaneous urticaria. In this trial, oral miltefosine medication was, depending on tolerability and timing in the 4 weeks treatment protocol, 0.6, 1.2 or 1.8 mg/kg/daily^16^.

### Prophylactic miltefosine treatment in a rat model of intracolonic capsaicin

Investigations in the maternal separation model and IBS patients showed the relevance of the TRPV1 cation channel for visceral hypersensitivity^6,10^. In an earlier investigation, we used the specific TRPV1-antagonist SB-705498 to show that intracolonic capsaicin-induced visceral hypersensitivity is strictly TRPV1 dependent^24^. Here we used the same capsaicin model to address the possible TRPV1 modulating capacity of miltefosine. After performing a baseline distension protocol at day 0, miltefosine (10 mg/kg) or vehicle were administered daily per oral gavage. A second distension protocol was then carried out at day 6. Subsequently, capsaicin (Sigma-Aldrich, St Louis, MO, USA) was administered under short isoflurane anesthesia. First we applied Vaseline (Boots Healthcare, Hilversum, The Netherlands) to the perianal area to avoid stimulation of somatic areas. Next, 100 µL 0.1% capsaicin (dissolved in 10% ethanol, 10% Tween-80, 80% saline) was given through a fine cannula with a rounded tip inserted rectally, 2 cm from the anus. Animals were allowed to recover for 90 min, after which the last distension protocol was performed. A schematic representation of the intracolonic capsaicin protocol is given in Fig. 4D.

### DNA extraction

In our previous investigations we compared the fecal mycobiome of nonhandled and maternally separated rats and observed profound differences between groups^4^. In the present investigations we compared the myco- and microbiome of maternally separated rats treated with either vehicle or miltefosine (10 mg/kg daily). DNA was isolated from fecal pellets that were collected directly from the anus on day 8 of the treatment protocol and stored at −80 °C until use. Importantly, isolation of fungal/yeast DNA requires more harsh methods than isolation of bacterial DNA. We used a lyticase-based method to catalyze fungal cell lysis, exact details of which are described earlier^4^. Isolated DNA was then used for ITS-1 as well as 16S sequencing.

### Fungal internal transcribed spacer (ITS) regions sequencing and visualization of results

Preparation of fungal amplicons was performed as described previously^4^. In summary, a two-step PCR was designed: the first PCR amplified ITS-1 regions, the second PCR generated Nextera XT tagmentation-compatible ITS-1 fragments^40^. These PCR products were additionally amplified (10 cycles, T_a_ = 49 °C). Reaction products were cleaned, and 8 bp Illumina sequencing adapters were ligated according to the manufacturer’s protocol (Nextera XT Index Kit; Illumina, San Diego, CA). Subsequently, barcoded products were quantified, and pooled at equal concentrations. After gel purification (Qiaquick spin kit; Qiagen), pooled samples were sequenced (250 bp paired-end sequencing, MiSeq; Illumina).

Raw fastq files were processed by demultiplexing, quality fiterling, and further analysed using Mothur and implanted modules^41^. The RDP-II Naïve Bayesian Classifier was used to taxonomically classify unique sequences^42^, using a 60% confidence threshold against the UNITE database (v7)^43^. The Bray-Curtis dissimilarity index was used for classical clustering (UPGMA algorithm) to assess and visualize distances between ITS compositions. PAST (v3.034) was used to generate nun-metric multidimensional scaling plots. One-way permutational multivariate analysis of variance (PERMANOVA) was done on the resulting Bray-Curtis dissimilarities. All raw sequencing data will be uploaded in the European Nucleotide Archive.

### Bacterial 16S sequencing and visualization of results

The hypervariable V4 region of the 16S-rRNA gene of the rat fecal DNA was amplified, sequenced by Illumina MiSeq (Illumina Inc., San Diego, CA, USA) and processed using modules implemented in the Mothur software platform, version 1.31.2 and Btrim^41,44^. First, reads were checked and quality trimmed (quality threshold 30) by using the ‘Btrim’ command. Next, read pairs were merged (‘make.contigs’), and merged reads with a length of 240–260 base pairs were aligned (‘align.seqs’) to a SILVA reference database. Visualization based upon Bray-Curtis dissimilarity was carried out analogous to ITS visualization described above.

### Agar disk diffusion assay to address antifungal and antibacterial properties of miltefosine

The antifungal and antibacterial qualities of miltefosine, were assayed at 4 different concentrations (50, 250 and 1000 µM and 10 mM). Methodology was similar to our earlier publication^31^ and is given in short only. To test fungicidal activity, *C*. *albicans* was diluted in top agar, spread in petri dishes and left to solidify. Next, 3 mm holes were punched and filled with 8 µl miltefosine, vehicle or the fungicide nystatin that was used as positive control. Plates were incubated 24 to 48 hour before the halo of inhibition, visible as a zone of clearing around a punched hole, was measured (diameter in mm). To test bactericidal activity, *B*. *subtilis* was diluted in 0.3% TBS/1% Agar/0.02% Tween, spread in petri dishes and left to solidify. Next, 3 mm holes were made and filled with miltefosine, vehicle or positive control (penicillin/streptomycin mixture containing 1000 units/ml and 1000 µg/ml respectively). After diffusion into the plates, dishes were covered with a top layer and further incubated for 18 to 24 hours, after which the halo-diameter was measured.

### Semi quantitative assessment of *in situ* mast cell activation status

Mucosal mast cells were stained following a staining protocol described by Wingren and Enerbäck^45^. As the distension took place in the distal part of the colon, paraffin sections were cut from proximal colon (thickness 4 μm) in order to rule out any role for distension-induced mast cell degranulation. Sections were processed for staining and incubated for 6 days in Toluidine Blue in 0.5N HCL (pH = 0.5). To semi-quantitatively assess mast cell activation, every 5th section, with a total of 5 sections per rat, was evaluated in a double-blind manner. A total of 50 mast cells were counted per section and each mast cell was categorized as either dark staining intensity, medium staining intensity or light staining intensity (example stainings are shown in Fig. 3A). Results of the 5 individual sections were averaged per rat to give a representative outcome. Final results are given as a percentage per category of total mast cells counted.

### Assessment of *in vitro* TRPV1 activation and modulation, by fluorimetric measurement of intracellular free calcium

Capsaicin-induced activation of TRPV1 and modulation thereof was monitored with the help of the Indo-1 AM calcium indicator (Invitrogen, Bleiswijk, the Netherlands). In short, TRPV1 recombinant SH-SY5Y human neuroblastoma cells (SH-SY5Y_hTRPV1_, kindly provided by GlaxoSmithKline, Stevenage, UK)^46^ were detached with the aid of trypsin free cell dissociation buffer (Gibco, Bleiswijk, The Netherlands), resuspended in HEPES-buffered HBBS and incubated with 10 μg/ml Indo-1 AM (30 minutes, 37 °C). Next, cells were washed and rested (30 minutes, room temperature) to allow complete de-esterification of intracellular esters. Hereafter, cells were re-suspended to 10^7^ cells/ml, supplemented with 1.2 mM CaCl_2_ and allowed to adapt to 37 °C for 10 minutes. Miltefosine (50, 70, 100 µM) was added 10 minutes before stimulating cells with capsaicin (32 nM, Sigma-Aldrich, St Louis, MO, USA). Similar to our earlier investigations^6,24^, we used the selective TRPV-1 antagonist SB-705494 (5 µM, kind gift of GlaxoSmithKline)^27^ as positive control for TRPV1 inhibition. Optimal dosage of capsaicin was first established using wildtype SH-SY5Y and SH-SY5Y_hTRPV1_ cells. Analyses were performed with NOVOstar analyzer (BMG Labtech GmbH, Offenburg, Germany; excitation, 320 nm; emission, 405 nm and 520 nm). Cytosolic free calcium/calcium influx is represented as the change in fluorescence at 405 nm divided by that at 520 nm = Δ405/520.

### Statistical analysis

Statistical analysis was performed using GraphPad Prism (version 7.03, Graphpad software, San Diego, USA). All data, excluding myco- and microbiome compositions, were tested for normality using D’Agostino & Pearson normality test. Visceromotor response data were analyzed with the Repeated Measures one-way ANOVA, and tested post-hoc with Sidak’s multiple comparisons test. Anti-microbial activity and calcium measurement data were analyzed with one-way ANOVA and Dunnett’s multiple comparisons post hoc test. Toluidine Blue staining intensity data were analyzed with the Mann Whitney U test, comparing the percentages of mast cells in each group.
